# The eyes as the exclamation mark of the face: exploring the relationship between eye size, intensity of female facial expressions and attractiveness in a range of emotions

**DOI:** 10.3389/fpsyg.2024.1421707

**Published:** 2024-08-08

**Authors:** Alanís Esté Jaloveckas, Roser Granero

**Affiliations:** ^1^Faculty of Psychology, Universitat Autònoma de Barcelona - UAB, Barcelona, Spain; ^2^Department of Psychobiology and Methodology, Universitat Autònoma de Barcelona - UAB, Barcelona, Spain

**Keywords:** emotion intensity, emotion perception, facial expression, eye size, avatar generation

## Abstract

**Background-objective:**

The eyes play an important role in communicating emotions and shape the determination of other facial attributes. Here, we explored the relationship between eye size, perceived intensity and attractiveness of facial expressions.

**Methods:**

A sample of *N* = 63 participants (men and women, aged 18–35) rated attractiveness and emotional intensity for images displaying emotionally expressive women’s faces with digitally manipulated eye size (15% smaller, unchanged, or 15% larger).

**Results:**

The analysis of perceived intensity showed an interaction parameter between eye size and gender. Female individuals reported differences when comparing unchanged and larger eyes; male participants showed differences across all eye size comparisons (smaller-unchanged, smaller-larger, unchanged-larger). Regarding perceived attractiveness, faces with smaller eyes registered lower mean scores than both unchanged and larger. The lowest intensity level was associated with neutral faces and the highest with fearful ones. Faces displaying happiness were perceived as the most attractive.

**Conclusion:**

Larger eyes seem to make emotions more intense and attractive. We suggest that the more intense phenomenon serves an evolutive purpose, as it might encourage caretaking behavior.

## 1 Introduction

Ekman and Friesen (1975) stated that when wanting to assess the truthfulness of someone’s expression, the feature that should be primarily paid attention to is the eyes. Baron-Cohen et al. (2001) suggested that a person’s state of mind can be decoded by looking at the widening and narrowing of the eyes. Lee and Anderson (2017) endorsed the idea that the eyes’ area reveals emotional states without the need to observe other parts of the face; and that no other facial feature reveals as much information about emotional processing as the eyes. Current studies have also highlighted the fact that facial expressions, including the perception of eye size, can serve as salient nonverbal signals to capture individuals’ attention (Carlson and Aday, 2018; Aday et al., 2023).

Conversely, Eisenbarth and Alpers (2011) observed that, when initially looking at a face, the first gaze tends to be directed toward either the eyes or the mouth. Blais et al. (2012) advocated that the most distinct facial gestures across expressions are found in the mouth area, and that this region provides the most relevant cues to recognize facial expressions. Salience is a key factor in faces with a smile, independently of the eyes (whether or not congruent) that accompany the mouth (Fernández-Martín et al., 2013; Calvo et al., 2016). Carbon (2020) proposed that not seeing the lower half of the face (the mouth and surrounding area) impairs emotional recognition to the point of misinterpreting happy, sad, or angry expressions as neutral.

Eisenbarth and Alpers (2011) addressed the question by subdividing it into specific emotions. According to these authors, sadness and anger are mostly noticed in the eyes. Attention is mainly directed toward the mouth in happy faces; and in fearful and neutral expressions, the eyes and mouth are equally important. Paradoxically, Barrett (2018) indicated that a clear indicator of fear are widely open eyes. Ekman and Friesen (1975) argued that, although fear is initially shown in the eyebrows, its intensity is expressed by the openness of the eyes. Notably, when contradictory stimuli are shown (i.e., happy mouth and sad eyes simultaneously, or vice versa), people choose to rely on the mouth (Dunlap, 1927). Recent studies have compared emotions in faces with and without surgical masks. Langbehn et al. (2022) showed that, when the mouth is hidden, the information about the emotion on the face shrinks. Tsantani et al. (2022) supported the idea that the intensity of facial expressions is reduced in all basic emotions—joy, fear, anger, sadness, disgust, and surprise (Ekman et al., 1969; Jack et al., 2012)—except anger, when the mouth is hidden. Kim et al. (2022) observed that, when compared with the eyes, the mouth was most salient for happiness, sadness, and anger; however, in the case of fear, the eyes achieved higher relevance.

Succinctly, empirical research suggests that both eyes and mouth are important for emotional decoding; that the information drawn from the face varies depending on the emotion; and that attention is paid to one or another part of the face depending on the emotion on display. Furthermore, it has been hypothesized that it is not the components of the face, but rather the areas (i.e., the center), that attract our attention. However, the more familiar the face, the higher the chance that attention will be drawn to the eyes (Royer et al., 2016). Other components of the face also claim visual attention [e.g., eyebrows are fundamental in anger (Ekman and Friesen, 1975)]. Therefore, it is not a simple eye vs. mouth question for all emotions.

Rather than analyzing which components or facial areas provide more information, some studies have focused on how those features work together in the face (Bartlett and Searcy, 1993; Xiao et al., 2014; Matsushita et al., 2015a). It has been suggested that faces could be recognized in a holistic way (Maurer et al., 2002) (as a unit rather than as a collection of components). However, emotions could be interpreted in different ways. For example, Tanaka et al. (2012) suggested that emotion recognition should be holistic when information is contradictory (such as a smiley face with angry eyebrows), but divided into different elements when all components point in the same direction (i.e., a happy face). Contrarily, Omigbodun and Cottrell (2013) argued that facial expression processing is essentially holistic, independent of the components. Curby et al. (2012) proposed that differences in the processing type are associated with the emotion types, negative emotions being less globally processed. Regarding the rating of attractiveness, facial proportions have been suggested to play a fundamental role in the holistic assessment of beauty in women (Ulrich et al., 2019).

Studies have addressed the relationship between eye size, perceived level of attractiveness and perceived intensity of emotions (Reis et al., 1990; Oosterhof and Todorov, 2009; Schmidt et al., 2012; Golle et al., 2013; Przylipiak et al., 2018). Regarding the three possible analyses (association between eye size and attractiveness, eye size and intensity, and attractiveness and intensity), research has mainly focused on the impact of eye size on attractiveness, based on the potential evolutionary advantage of larger eyes (increased eye size has been associated with improved visual perceptiveness, a key capacity for finding food and detecting predators in animals). Berry and McArthur (1985) proposed that large eyes, high eyebrows, and narrow chins generate baby-like appearances. Glocker et al. (2009) suggested that babies’ eye size is attractive due to evolution and that cuteness motivates caretaking behavior, which increases the child’s chances of survival. Larger eyes have also been linked to attractiveness in adulthood (Berry and McArthur, 1985). Although averageness has repeatedly been linked to attractiveness (Thornhill and Gangestad, 1993; Grammer and Thornhill, 1994; Scheib et al., 1999), larger eyes might signify health, which is an influential factor when choosing a sexual mate (Thornhill and Gangestad, 1999). When other parts of the face (e.g., nose, lips) are smaller—making the eyes more dominant—attractiveness rates increase (Przylipiak et al., 2018). On the flip side, effects that make the eyes more attractive have been shown to make them seem larger (Matsushita et al., 2015b). These studies suggest that eye size also has an influence on attraction in adulthood. Noticeably, the relationship between attractiveness and eye size has been analyzed using essentially positive emotions, mostly happiness (Wagner, 1990), and it has been observed that faces characterized by positive emotions are perceived as more attractive than faces with neutral or negative expressions (Reis et al., 1990; Oosterhof and Todorov, 2009; Schmidt et al., 2012; Golle et al., 2013; Ueda et al., 2016).

The link between attractiveness and intensity has only been found to be significant when positive emotions are considered. The study by Golle et al. (2013) observed that happier faces were perceived as more attractive than faces reflecting other emotion types. Another study aiming to compare the intensity and attractiveness levels of faces with happy, sad, and neutral expressions observed that more intense happy faces were considered more attractive than less intense happy faces (Ueda et al., 2016). This work also observed that sad faces evaluated as more intense were not perceived as less attractive. This evidence suggests that positive and negative emotions do not have opposite effects on the perception of attractiveness. Other studies also obtained higher intensity levels associated with angry and happy faces, as compared with neutral faces (Garrido and Prada, 2017).

Studies have also explored the role of the participants’ gender when interpreting emotion intensity assessments of facial expressions. Hama and Koeda (2023) suggested that female participants rated happy and sad expressions as more intense than male individuals. Happiness has also been identified as more intensely perceived among women rating female images, and less intensely when men are rating male images. Gong et al. (2018) have also suggested that females perceive both positive and negative emotions as more intense than males. However, these results also seem to be associated with age and culture. The aforementioned studies were carried out in Japan and China. A study carried out with German participants (Gong et al., 2018) suggested that women perceive negative emotions more intensely and positive emotions less intensely, when compared to men. Regarding attractiveness levels, studies suggest that this measure may be similar regardless of participants’ gender (Kranz and Ishai, 2006; Levy et al., 2008).

In summary, empirical research provides inconsistent results about the role of the eyes in the recognition of emotions. A number of studies support the theory that the eyes provide cues to the emotional state of people, thus claiming the main role in the recognition of emotions in the face (Ekman and Friesen, 1975; Baron-Cohen et al., 2001; Lee and Anderson, 2017). Other findings suggest that the eyes are not always the main values of emotion, but that this role is instead claimed by the mouth (Dunlap, 1927; Blais et al., 2012; Carbon, 2020; Langbehn et al., 2022). Either way, this approach sustains that eyes could be a central element (primary or secondary, in addition to the mouth) for decoding and assessing emotional states, and that the specific role of the eye could vary depending on the emotion type (Eisenbarth and Alpers, 2011; Kim et al., 2022; Tsantani et al., 2022). An alternative theory proposes that emotions, like faces, are processed in a holistic way, rather than by separate components (Omigbodun and Cottrell, 2013). However, results in this research area are contradictory (Curby et al., 2012; Tanaka et al., 2012), suggesting that although some features of the face could be relevant in understanding specific emotions, the face as a whole could also have effects on emotional decoding.

Previous studies have also established a relationship between perceived attractiveness and intensity of emotions, as well as between attractiveness and eye size. It must be stressed that the link between intensity and eye size remains mostly unexplored, and some results seem inconsistent and related to the emotions type. Overall, eye size ratings have been shown to increase alongside attractiveness, and apace with the intensity of happiness (Ueda et al., 2016). Fewer studies have addressed negative emotions. Regarding attractiveness, negative emotions are perceived as less attractive than happy and neutral ones (Ueda et al., 2016; Garrido and Prada, 2017). Regarding intensity, Eisenbarth and Alpers (2011) argued that eyes achieve a more central role than the mouth for the assessment of angry and sad faces, which in turn might make bigger eyes distort the perception of intensity in those emotions. Moreover, fearful and neutral faces are understood equally from the eyes and the mouth, which reduces the role of the eyes in comparison to the other emotions. Paradoxically, fearful faces are characteristically recognized by widened eyes (Barrett, 2018), which suggests the relevance of the eyes when it comes to this emotion. Gender and age might also have an effect on intensity (Gong et al., 2018; Hama and Koeda, 2023). Participant’s gender and sexual preference should also be considered when interpreting attractiveness ratings (Kranz and Ishai, 2006; Levy et al., 2008).

Additionally, when human representations are artificially altered to diverge from reality, it is essential to consider the degree of these changes. The uncanny valley is the phenomenon in which artificial human representations are perceived as uncomfortable as they become more similar to reality (Mori et al., 2012). This phenomenon is commonly seen in video games, robotics, and human-like dolls (Cheetham, 2011).

To summarize, there is debate concerning to what extent emotions are perceived in the eyes. Empirical results suggest that eye size could be a key indicator of some emotions, and that this characteristic might modify the intensity of the emotional cues provided by the face as a whole. Concretely, larger eyes would make happy faces look happier, angry faces angrier, and so on. According to evolution-based theories, neutral faces with bigger eyes may also be rated as more intense than those with smaller eyes (Berry and McArthur, 1985; Glocker et al., 2009). The hypothesis that eye size intensifies facial emotional clues also supports the argument that emotions are processed holistically, and that emotional expressions are deciphered by understanding the face as a compendium of pieces that must be looked at together, as the emotions in which the eyes do not have as much of an effect (e.g., happiness) would still be affected by the face in its entirety. However, the results are uncertain and new empirical studies are needed to consolidate (or refute) this line of research.

The main goal of the present study was to examine the relationship between eye size and facial emotional expression with the perceived intensity and attractiveness ratings. In addition, the study explored the potential moderator role of gender and age within the aforementioned relationships. Based on the existing empirical studies, we hypothesized that larger eyes should be associated with higher scores in attractiveness and intensity, particularly for fear and happiness. Due to the lack of previous empirical evidence, no hypothesis was formulated regarding the potential interaction of gender and age in the relationships.

## 2 Materials and methods

### 2.1 Sample

The experiment was shared as a URL. Participants were recruited with a short text message introducing them to the study via social media (e.g., Instagram) and online chats (e.g., WhatsApp). The URL was further spread by means of snowball sampling. Participants volunteered their time. There were no economic or otherwise relevant incentives. Inclusion criteria for all participants were: to have normal or corrected to normal vision and to be between 18 and 35 years of age. No participants were removed due to gender, age, ethnicity, or occupation.

An initial sample of *N* = 82 individuals started the experiment. Nineteen subjects did not complete the first section with the demographics or abandoned during the training task, which led to a final sample consisting of *N* = 63 participants (43 were classified as *Female*, 17 as *Male* and 3 as *Other*) who completed the experimental task. The task was not only repetitive but also long to complete. We hypothesize it required too much time and some participants lost motivation to continue. Those two aspects might have led the 19 subjects to abandon the experiment before completing it.

**Figure 1:**
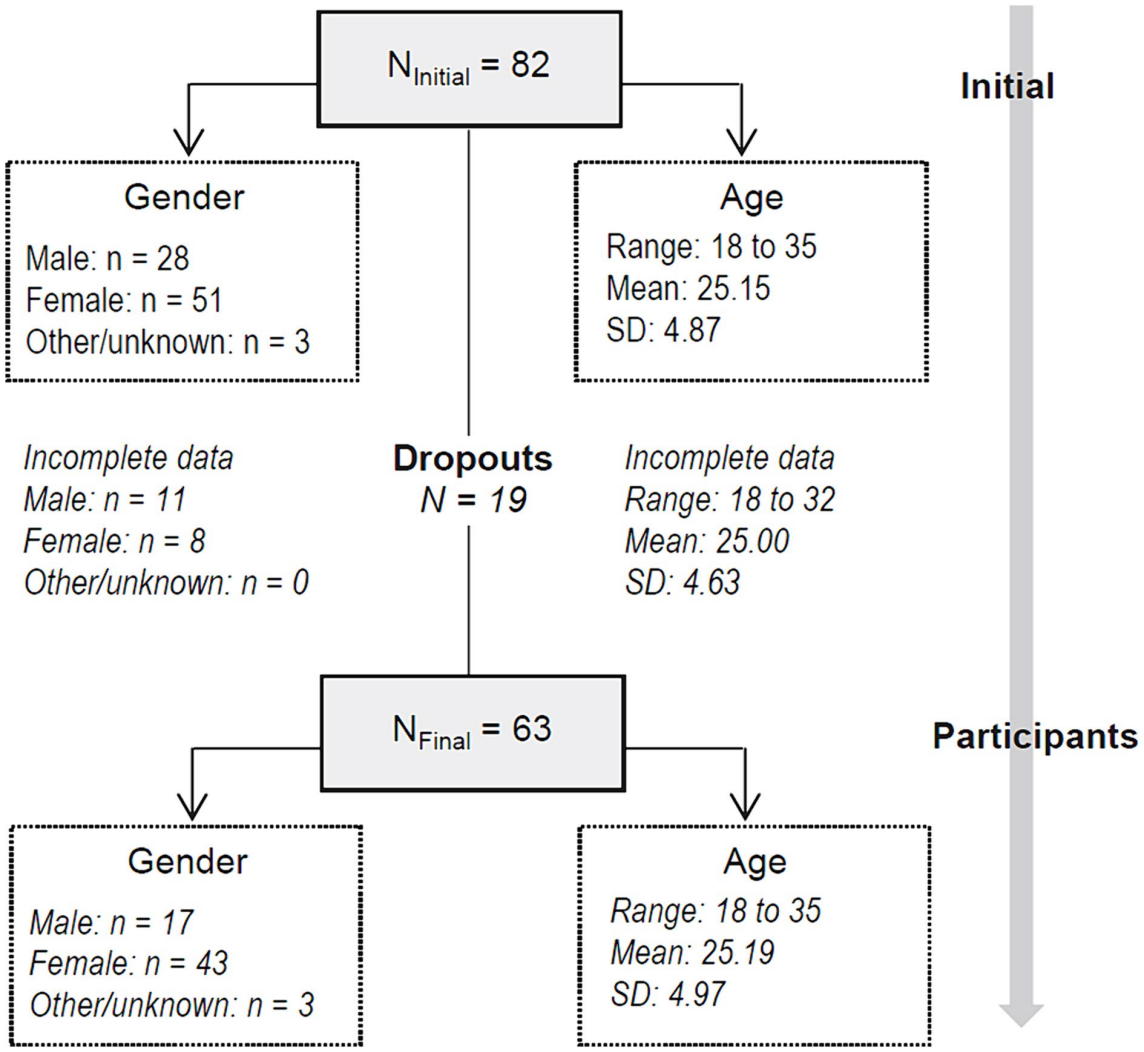
Flow-chart with the sampling method.

Figure 1 shows the flow-chart with the sampling process (participants retained during the experimental task and dropouts). No statistically significant differences between completers and dropouts were observed for gender (*p* = 0.051), age (*p* = 0.082), employment status (*p* = 0.264), and ethnicity (*p* = 0.440).

### 2.2 Materials

#### 2.2.1 Stimuli

To conduct the study, some of the previously defined basic emotions were used: anger, joy, fear, and sadness. Not all original Ekman’s facial expressions—joy, fear, anger, sadness, disgust, and surprise (Ekman et al., 1969; Jack et al., 2012)—were selected for the experimental task, as doing so would have required more time to complete it, and a longer task might have increased the number of dropouts. A neutral expression was used to set a baseline for the intensity ratings. The emotions chosen for this study were selected based on those that have received the most attention in previous scientific research. Some of the studies addressing the topics of intensity, attractiveness or eye size in relation to decoding facial expressions have used either the exact same emotions used in this study (Eisenbarth and Alpers, 2011) or a subset of them (Oosterhof and Todorov, 2009; Carlson and Aday, 2018; Aday et al., 2023).

Thirty-five pictures—seven female identities, each displaying five facial expressions: neutral, happiness, fear, sadness and anger—were selected from *The racially diverse affective expression (RADIATE) face stimulus set* (Conley et al., 2018). The code-files used for the experimental trials were faces AF01, AF12, BF03, BF09, WF06, and WF10; and the code-file used for the training trial was face WF15. All the images corresponded to female identities spanning a mixture of ethnicities (Asian, Black, White). There were two female faces of each ethnic group. Male identities were not included with the purpose of reducing the time needed to complete the experiment, as the inclusion of a new experimental condition (male faces) would have doubled the time needed to complete the experiment, thus increasing the chances that a larger number of participants abandoned the study, as they received no monetary or otherwise compensation for their participation. Furthermore, the consideration of this new experimental condition would have required the inclusion of a new experimental factor, and the planned sample size might not have allowed adequate statistical power. Female faces were chosen over male ones because it is more common to use male identities, rather than female, in psychological experiments; doing it differently adds representativity to the field. Faces with hair in front of the eyes in any expression were discarded, as this would have complicated the process of digitally altering the eye size within the stimuli.

**Figure 2:**
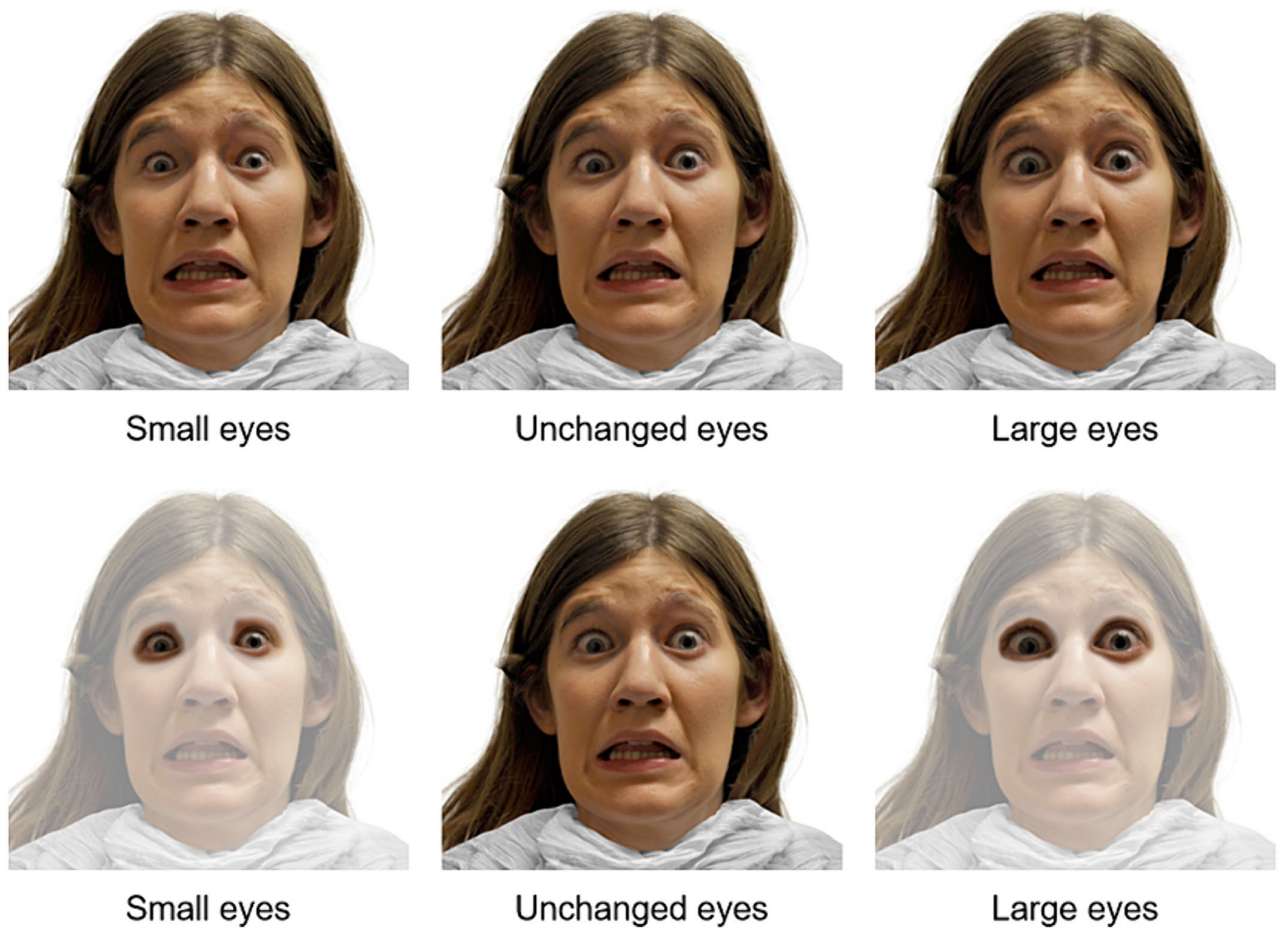
Eye sizes on a scared face used during the experimental trials.

Three versions of each original picture were used in the experiment. The original, unmodified image was used for the medium-sized eyes. Previous research has worked with values of 5–10% change (Glocker et al., 2009) in similar experiments. With the purpose of assessing a different degree of change than the ones already studied and published by the scientific community, the eyes were changed 15% from their original size. Each picture was modified twice. The eyes were reduced 15% for the smaller eye images and increased 15% for the larger ones (including eyelids and eyelashes, excluding eyebrows). The eyes area was enlarged or reduced as necessary, and then blended into the picture so that the change was not obvious. To edit the images *Adobe Photoshop* version 16.0 was used. See Figure 2 for examples of images used during the experiment with different eye sizes, as well as an example of how the eyes area was modified. The original unchanged images are part of the open-access RADIATE face stimulus set published by Conley et al. (2018).

Fifteen images of one female identity (five expressions, three eye sizes) were readied to be used only during an initial training task. Ninety images of the remaining six female identities (five expressions, three eye sizes, each) were readied to be used during the experimental task. The identities used for the practice and experimental trials remained constant for all participants.

The experiment was built and conducted using Sona Systems written in PHP and JavaScript, using MySQL for the database. Demographics and experimental data were collected online with participants able to undertake the task on any computer/laptop but not tablet or smart device.

### 2.3 Data collection

Five fixed lists of stimuli were created for the experimental trials. Participants were randomly assigned to one of the lists when they started the experiment. The order of the images was randomized beforehand for the purpose of facilitating the technicalities of setting up the experiment. Images with the same emotion and human face were not shown twice in a row during the trials so that the difference in eye sizes was not apparent. MATLAB software and in-house code were used to create the random list of images, with the condition that images of the same identity and expression with different eye size did not follow each other. The experimental trials were broken down into three equally long blocks with self-paced short breaks in between to avoid tiredness. During the breaks an encouraging message was shown on the screen accompanied by a picture of an animal. One main reason for dropping out of experimental studies is a lack of motivation, particularly in studies with no financial compensation for participation. The message was meant to keep participants engaged in the experimental task with the purpose of reducing the number of dropouts. The message was also meant to let participants know it was the moment to take a pause should they need it. Additionally, the message indicated how far along in the experiment they had arrived (1/3 the first message and 2/3 the second message). The purpose of the picture was to make the experiment less mentally tiring.

Participants were naive to the experimental tasks and purpose. The entire experiment was carried out in approximately 20 min. Firstly, participants were shown a welcoming message that introduced them to the study. Next, they were asked to complete the demographic section, and a training task to ensure that they adequately understood how to perform the experimental task. Participants were instructed to complete both the practice and experimental tasks in a quiet space with stable internet connection, and to maximize their browser.

There were a total of 95 trials (including the five training trials). Each training trial displayed one of the five emotions (neutral, happiness, fear, sadness, anger), with one of the eye sizes. On all trials, participants evaluated each face twice, rating first the intensity of the emotion shown, and then the attractiveness of each face, on a scale from 1 to 7. During the trials the images were presented in color, placed in the center of the screen and covered approximately 40% of the screen space. A written question shown below each image informed the participant of the task: (1) *How intense do you find her expression?* (2) *How attractive do you find her?* The image remained on screen until both evaluations had been made. The order of questions was fixed. Participants responded based on a seven-point Likert scale from 1 (lowest intensity level: *Not very intense*/*Not very attractive*) to 7 (highest intensity level: *Very intense*/*Very attractive*) located below the question. Participants were only informed of the interpretation of the extremes of the scale (values 1 and 7), similar to an analog scale. Each image was preceded by a fixation cross for 500 milliseconds, and subsequent trials began immediately after the second response.

After the training task, participants moved on to the experimental task. There were six experimental trials per condition. Only female faces were selected for the experiment. The experimental task consisted of 90 trials split into three equal blocks. Breaks were self-paced and featured a message (e.g., “*End of part 1 (of 3), you are doing great!*”) and an accompanying encouraging picture. The procedure followed the same structure as in the training trials. Participants were shown one image at a time. There was no time limit to rate the images. Once finished, participants were debriefed and thanked for participating (Supplementary Table S1).

Participants rated the intensity and attractiveness of 90 images, that corresponded to 6 different human faces presented in 15 experimental conditions (three eye sizes × five emotions). The statistical analysis was performed for the mean value calculated for the 6 human faces in each experimental condition. That is, each participant provided 15 measures of the attractiveness level and 15 measures of the intensity level (for the combination of the three eye sizes with the five emotion types).

### 2.4 Statistical analysis

The statistical analysis was carried out with SPSS24 for Windows. Repeated measures analysis of variance (ANOVA) was used to assess the effect of eye size (within-subjects factor with three levels: small, unchanged, large) and emotion type (within-subjects factor with five levels: neutral, happiness, fear, sadness, anger). To assess the potential moderator effect of the participants’ gender (female, male) and age (young, middle), two between-subjects factors were also added into the model and tested, resulting in a 3 × 5 × 2 × 2 ANOVA (eye × emotion × gender × age). The dependent variables of the analyses were the perceived intensity and attractiveness of the visualized emotions.

Mauchly’s test was used to assess the sphericity condition for the ANOVA procedures, and if the assumption was not met (*p* < 0.05) Greenhouse–Geisser corrected tests were selected. The effect size of the parameters obtained in the ANOVA was measured through partial eta-squared coefficients (η*_p_*^2^), considering values of 0.06 as poor, 0.10 as moderate–mild and 0.25 as high-large (Levine and Hullet, 2002). Because of the relatively small sample size (that could have had an impact on the statistical power), relevant effects were considered for both any statistical significance (*p* ≤ 0.05) or effect sizes at least in the moderate–mild range (η*_p_*^2^ ≥ 0.01).

### 2.5 Ethical considerations

The experiment was approved by the ethics board of the Department of Psychological Sciences, Birkbeck College, University of London (approval number: 2122068, date of approval: 09/05/2022). All the participants provided informed consent by ticking a box within the website before starting the experiment. Participants did not receive economic compensation for their engagement in the research.

## 3 Results

### 3.1 Descriptive for the sample

Table 1 shows the descriptive for the sample. Most participants were female (68.3%), white-European (81.0%), and employed (44.4%). Mean age was 25.2 years (SD = 5.0). No differences between female and male participants were obtained for ethnicity (χ^2^ = 0.42, *p* = 1.00), employment status (χ^2^ = 2.26, *p* = 0.447) and chronological mean age (*F* = 0.99, *p* = 0.377).

**Table 1:** Descriptive for the sample.

	*n*	%
Gender		
Female	43	68.25%
Male	17	26.98%
Other	2	3.17%
Missing	1	1.59%
Ethnicity		
White – European	51	80.95%
Other	10	15.87%
Missing	2	3.17%
Employment		
Employed	28	44.44%
Student	27	42.86%
Missing	8	12.70%
	Mean	SD
Aye (years-old)	25.19	4.97

SD, standard deviation.

Table 2 contains the descriptive for both the intensity and the attractiveness levels in the study, among the total sample and stratified by participants’ gender and age (classification into the two age groups was based on the median [percentile 50] estimated in the sample). Supplementary Figure S1 shows line-plots with the mean scores for both intensity and attractiveness, considering the within-subjects factors (eye size and emotion type) and the between-subjects factors (gender and age).

**Table 2:** Descriptive for intensity and attractiveness levels.

			Emotion									
			Neutral		Happiness		Fear		Sadness		Anger	
Total sample	*N* = 63		Mean	SD	Mean	SD	Mean	SD	Mean	SD	Mean	SD
Intensity	Eye	Small	2.11	1.05	4.67	1.09	5.78	0.72	4.18	1.01	4.84	0.79
		Unchanged	2.16	1.05	4.79	1.03	5.77	0.67	4.18	0.88	4.83	0.86
		Large	2.37	1.07	4.85	1.13	5.98	0.74	4.33	0.91	5.03	0.89
Attractiveness	Eye	Small	2.29	1.01	3.25	1.35	2.36	0.98	2.36	0.92	2.51	0.94
		Unchanged	2.92	1.07	3.90	1.40	2.74	1.00	2.78	0.96	2.79	1.02
		Large	2.81	1.01	3.79	1.23	2.63	0.95	2.72	1.00	2.83	1.00
Female	*N* = 43		Mean	SD	Mean	SD	Mean	SD	Mean	SD	Mean	SD
Intensity	Eye	Small	2.05	1.08	4.67	1.21	5.80	0.75	4.18	1.12	4.83	0.91
		Unchanged	2.03	1.02	4.77	1.05	5.74	0.70	4.16	0.98	4.79	0.96
		Large	2.17	0.97	4.74	1.17	5.96	0.74	4.22	0.94	5.00	0.95
Attractiveness	Eye	Small	2.30	1.11	3.28	1.40	2.43	1.08	2.33	0.98	2.51	1.04
		Unchanged	2.90	1.15	3.93	1.41	2.78	1.08	2.82	1.07	2.80	1.16
		Large	2.81	1.03	3.74	1.24	2.64	1.01	2.70	1.10	2.82	1.10
Male	*N* = 17		Mean	SD	Mean	SD	Mean	SD	Mean	SD	Mean	SD
Intensity	Eye	Small	2.27	1.03	4.75	0.60	5.70	0.69	4.18	0.77	4.82	0.42
		Unchanged	2.46	1.13	4.92	0.80	5.86	0.62	4.29	0.58	4.91	0.59
		Large	2.89	1.20	5.15	0.86	6.11	0.75	4.67	0.82	5.07	0.72
Attractiveness	Eye	Small	2.24	0.78	3.03	1.25	2.10	0.68	2.31	0.69	2.47	0.73
		Unchanged	2.93	0.91	3.72	1.45	2.59	0.86	2.65	0.67	2.76	0.66
		Large	2.83	1.04	3.78	1.21	2.58	0.80	2.72	0.78	2.76	0.70
Young age^a^	*N* = 31		Mean	SD	Mean	SD	Mean	SD	Mean	SD	Mean	SD
Intensity	Eye	Small	2.15	0.94	4.62	1.16	5.87	0.69	4.08	1.08	4.78	0.74
		Unchanged	2.14	0.93	4.75	1.17	5.77	0.64	4.08	0.96	4.75	0.82
		Large	2.25	0.93	4.82	1.24	5.96	0.78	4.20	0.87	4.99	0.88
Attractiveness^a^	Eye	Small	2.15	0.97	3.23	1.58	2.22	1.00	2.34	0.94	2.40	0.87
		Unchanged	2.71	0.99	3.79	1.58	2.46	0.96	2.56	0.88	2.57	0.91
		Large	2.61	1.02	3.71	1.44	2.46	0.88	2.56	0.95	2.67	0.94
Middle	*N* = 32		Mean	SD	Mean	SD	Mean	SD	Mean	SD	Mean	SD
Intensity	Eye	Small	2.06	1.15	4.72	1.02	5.70	0.74	4.24	0.95	4.86	0.87
		Unchanged	2.16	1.16	4.83	0.87	5.78	0.69	4.23	0.84	4.88	0.91
		Large	2.45	1.18	4.90	1.01	6.01	0.70	4.42	0.97	5.03	0.90
Attractiveness	Eye	Small	2.41	1.03	3.24	1.11	2.48	0.95	2.37	0.90	2.60	1.00
		Unchanged	3.08	1.13	3.95	1.23	2.99	0.97	2.98	1.00	2.98	1.09
		Large	2.98	0.98	3.83	1.00	2.77	0.99	2.83	1.05	2.95	1.04

a The groups of age (young and middle) were defined based on the median (percentile 50) in the study.

Young age: 18–25 years. Middle age: 26–35 years. SD, standard error.

### 3.2 Assessment of the interaction role of participants’ gender and age

The results of the initial ANOVAs assessing the interaction parameters between emotion and eye size with participants’ gender and age are displayed in Table 3. The second order interactions (emotion × eye size × gender, and emotion × eye size × age) were excluded from the ANOVA procedures based on the results achieved by these parameters (not statistically significant [*p* > 0.05] and poor effect size [η_p_^2^ < 0.10]). The participants’ age was also excluded from the ANOVA because the first order interactions (emotion × age, and eye size × age) also obtained non-statistical significance or irrelevant effect size. The between-subject factor was retained in the ANOVA that was carried out for the intensity level measure, based on the observed statistically significant interaction eye size × gender.

**Table 3:** Assessment of the first and second order interactions with participants’ gender and age.

	Emotion × gender			Eye size × gender			Emotion × eye size × gender		
	*F*	*p*	η*_p_*^2^	*F*	*p*	η*_p_*^2^	*F*	*p*	η*_p_*^2^
Intensity	0.776	0.493	0.013	5.417	0.010*	0.085	0.940	0.464	0.016
Attractiveness	0.278	0.726	0.005	0.647	0.497	0.011	0.911	0.495	0.015
	Emotion × age			Eye size × age			Emotion × eye size × age		
Intensity	0.213	0.871	0.003	0.625	0.501	0.010	0.779	0.586	0.013
Attractiveness	0.691	0.485	0.011	2.109	0.134	0.033	0.604	0.748	0.010

η*_p_*^2^: partial eta squared. *Bold: significant parameter.

### 3.3 Association between eye size and intensity

The ANOVA performed for intensity level scores included eye size (small, unchanged, and large) and emotion type (neutral, happiness, fear, sadness, and anger) as within-subjects factors, and participants’ gender (female, and male) as between-subjects factors. The complete results of this model are displayed in Supplementary Table S2.

The multivariate tests obtained non-statistical significance and an irrelevant effect size for the interaction emotion × eye size × gender (*F* = 0.94, *p* = 0.464, η*_p_*^2^ = 0.016), emotion × eye size (*F* = 0.66, *p* = 0.680, η*_p_*^2^ = 0.011) and emotion × gender (*F* = 0.78, *p* = 0.493, η*_p_*^2^ = 0.013). Statistically significant results were achieved for the interaction parameter eye size × gender (*F* = 5.42, *p* = 0.010, η*_p_*^2^ = 0.085). Based on these results, single effects were estimated for the eye size factor (that is, pairwise comparisons between small, unchanged, and large eye sizes were obtained, separately for female and male participants), and main effects were estimated for the emotion type factor.

**Figure 3:**
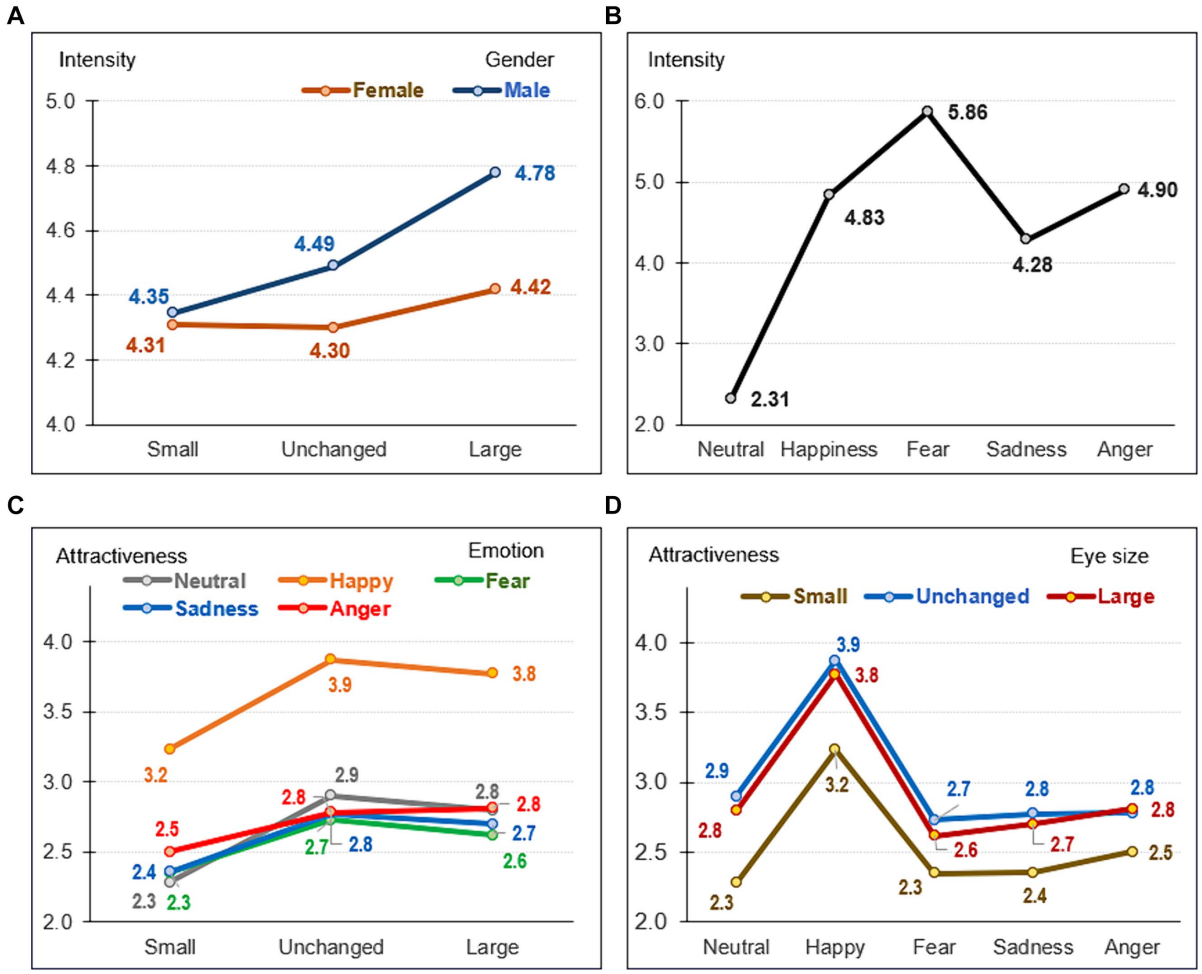
Visualization of the eye size effects on the intensity and the attractiveness levels. **(A)** Means for the intensity levels (Y-axis) based on the eye size (X-axis), stratified by the participants’ gender. **(B)** Means for the intensity levels (Y-axis) based on the emotion type (X-axis). **(C)** Means for the attractiveness levels (Y-axis) based on the eye size (X-axis), stratified by the emotion type. **(D)** Means for the attractiveness levels (Y-axis) based on the emotion type (X-axis), stratified by the eye size.

Figure 3A displays the graphic visualization of the interaction between eye size and participants’ gender. Among female individuals, differences were observed comparing unchanged eyes with larger eye size faces (*p* = 0.026). Among male participants, differences were identified across all comparisons (small versus unchanged: *p* = 0.026; small versus large: *p* < 0.001; unchanged versus large: *p* = 0.001). It is worth noting that the comparison between genders suggested that male (compared to female) subjects tended to report higher intensity levels for unchanged and large eye size faces.

Regarding the differences for intensity levels based on emotion type (Figure 3B), the lowest intensity mean score was associated with neutral faces and the highest with fearful ones. The contrasts between emotion types showed relevant differences across all pairwise comparisons (*p* < 0.001), except for the difference between happy and angry faces (*p* = 0.396).

### 3.4 Association between eye size and attractiveness level

The ANOVA obtained for attractiveness level included eye size (small, unchanged, and large) and emotion type (neutral, happiness, fear, sadness, and anger) as within-subjects factors. The complete results of this model are displayed in Supplementary Table S3.

Since the interaction parameter eye size × emotion reached statistically significant results (*F* = 4.17, *p* = 0.001, η*_p_*^2^ = 0.063), single effects were estimated and interpreted to assess the differences based on the eye size and the emotion type. Figure 3C displays the graphic visualization of this interaction effect. Regarding the eye size factor, all the pairwise comparisons between small eye size faces and unchanged and large reached relevant differences (*p* < 0.05), independently of the emotion. The mean comparisons between unchanged and large eye size faces showed no differences (independently of the emotion). For the emotion type factor, the highest attractiveness level was associated with happy faces across all eye size comparisons (Figure 3D).

## 4 Discussion

The aim of this study was to obtain empirical evidence regarding the association between eye size and the perceived intensity and attractiveness of different emotions, in an experimental task based on the visualization of female faces. The comparison of the intensity levels showed an interaction with participants’ gender: within the male participants, differences were found across all eye size comparisons. Within the females group, differences were only found when comparing the unchanged and larger eyes. No interaction was found between gender and emotion type. The emotion perceived as the most intense was fear, and neutral faces were rated with the lowest intensity. No interaction was found when comparing age by emotion and age by eye size. Regarding the perception of attractiveness, no significant interactions were found with either age or gender. The lowest mean was associated with the smaller eyes, with no differences between the unchanged and larger eyes. The most attractive emotion was happiness.

The results of this study are based solely on the visualization of images of female faces. Therefore, they should not be considered representative of or generalizable to both men and women.

Neutral expressions were expected to be perceived as the least intense (Garrido and Prada, 2017), and fearful ones the most intense (Ekman and Friesen, 1975; Becker, 2012; Żurowska et al., 2018). Happy and angry faces were expected to be equally intense, and sadness more intense than neutral, but less than the other three (Ueda et al., 2016; Garrido and Prada, 2017). The results of this study support these findings, and outline the premise that codification of face expressions is related to eye size. Moreover, participants’ gender reached a moderator role in the relationships.

The results of our study also suggest that attractiveness ratings are not dependent on the participants’ age or gender. However, differences have been found associated with the emotion type. We hypothesized that happy expressions should be rated as more attractive than all the others, and neutral more attractive than fearful, sad and angry (Golle et al., 2013; Ueda et al., 2016; Garrido and Prada, 2017). Some of these differences have been observed. Happy faces were more attractive than all the rest. Neutral, fear, sadness and anger were not rated as different from each other except for the comparison neutral-fear with large eyes, and neutral-anger with small eyes. In regard to attractiveness and eye size, bigger eyes were expected to be perceived as more attractive than smaller eyes (Glocker et al., 2009; Przylipiak et al., 2018). In particular, it was expected for large eyes to be rated as more attractive than unchanged and small. A difference was also anticipated between unchanged and smaller eyes, as previously found by Glocker et al. (2009). A main effect of eye size on attractiveness was replicated, but the difference was not found where it was expected: participants found smaller eyes to be less attractive than unchanged and larger; but there was no difference between unchanged and larger.

Overall, the results of this study suggest that emotions are perceived more intensely when the eye size is digitally increased in women’s faces, and that these changes are gender-dependent, men being more susceptible to changes than women. Noticeably, neutral faces with smaller eyes were rated as less intense than larger and unchanged (as the rest of emotions). This phenomenon—“more intense neutrality”—may suggest that larger eyes not only intensify emotions, but also increase the perceived intensity of the face in its entirety, which supports the theory that emotions are, at least partially, holistically perceived (Omigbodun and Cottrell, 2013), as bigger eyes intensify the message projected by the rest of the face.

Glocker et al. (2009) suggested that larger eyes in babies lead to a higher perception of attractiveness, which motivates caretaking behavior, thus increasing the child’s chances of survival. The phenomenon “the bigger the eyes, the more intense the emotion” supports the theory that emotions are perceived as more intense due to evolutive mechanisms: a sad baby with bigger eyes might seem sadder, which might prompt caregivers to take care of them more urgently. Both Glocker et al. (2009)’s attractiveness-caretaking behavior theory, and the theory “the bigger the eyes, the more intense the emotion” support the idea that bigger eyes increase the chances of survival by increasing both the intensity of the emotion on display, and the attractiveness of the face. This phenomenon is also applicable to adulthood age, as bigger eyes also make adult faces gain in attractiveness (Berry and McArthur, 1985; Thornhill and Gangestad, 1999; Matsushita et al., 2015b; Przylipiak et al., 2018) and intensity, as seen in this study.

The results of the present study support the premise that eye size has an impact on attractiveness: faces with larger eyes are generally perceived as more attractive than those with smaller eyes (Glocker et al., 2009; Przylipiak et al., 2018). However, our data also indicated that bigger than normal eyes do not enhance attractiveness any more than unchanged eyes. This is a novel finding, as previous literature has repeatedly suggested that bigger eyes are perceived as more attractive, especially in happy faces (Berry and McArthur, 1985; Glocker et al., 2009; Matsushita et al., 2015b; Ueda et al., 2016; Przylipiak et al., 2018; Hine and Okubo, 2021). In any case, our results should be interpreted considering that our research tested increase and decrease changes of 15%, while other experiments have tested smaller changes in the 5% or 10% range (Przylipiak et al., 2018). The modifications used in our study might have altered the facial expression too much making the images reach the uncanny valley, resulting in faces that were perceived as uncomfortable (missing any chance of gaining in attractiveness).

## 5 Limitations

The results of this work should be interpreted in accordance with certain limitations. First, the asymmetrical distribution of the participants’ gender (the proportion of male individuals was lower than female individuals) should be considered for generalization purposes.

Second, the female faces visualized in the experimental task showed characteristics in addition to the face area, such as hair, and therefore differences in attributes such as hairstyles could have affected attractiveness ratings due to individual preferences. Furthermore, the variety of faces in the experimental task was relatively low, which limited the possibility of including intra-ethnical differences. However, it must be considered that the high number of experimental conditions in our study (three eye size values multiplied by five emotion types) did not allow a greater number of images, otherwise the experiment would have been too long and tiring for the participants.

In relation to the previous point, the study was carried out using images of female faces. Other replication studies including male faces are necessary for generalization purposes, mostly because females are usually perceived as more attractive and/or intense than males (Garrido and Prada, 2017). Lastly, additional research that explores a wider range of emotions (including other primary emotions such as surprise or contempt) is necessary to further understand the role of eye size in the interpretation of facial expressions.

## 6 Conclusion

To our knowledge, this is the first study addressing the combined impact of eye size and emotions on the perceived intensity and attractiveness of facial expressions, considering the potential role of the participants’ gender and age. The results indicate that the eyes act as intensifiers of emotions, particularly among male respondents, and that facial emotional expressions are, at least partially, holistically perceived. The results of this work also show that the attractiveness ratings were affected by eye size, with smaller eyes perceived as less attractive, independently of the emotion. This evidence could be interpreted as an evolutionary phenomenon.

## Data availability statement

The datasets presented in this article are not readily available because restrictions apply to the datasets: limitations to making data publicly available include participant privacy, in accordance with the ethical consent provided by participants on the use of confidential/identifiable human data. Requests to access the datasets should be directed to AEJ, Alanis.Este@autonoma.cat.

## Ethics statement

The studies involving humans were approved by Ethics board of the Department of Psychological Sciences, Birkbeck College, University of London (approval number: 2122068, date of approval: 09/05/2022). The studies were conducted in accordance with the local legislation and institutional requirements. Participants provided informed consent by ticking a box within the website before starting the experiment. Written informed consent was obtained from the individual(s) for the publication of any identifiable images or data included in this article.

## Author contributions

AEJ: Conceptualization, Data curation, Formal analysis, Investigation, Methodology, Project administration, Writing – original draft, Writing – review & editing. RG: Formal analysis, Writing – original draft, Writing – review & editing.
