# Supplementary material for: The eyes as the exclamation mark of the face: exploring the relationship between eye size, intensity of female facial expressions and attractiveness in a range of emotions
Source: Front Psychol. 2024 Aug 8;15:1421707. doi: 10.3389/fpsyg.2024.1421707 (PMC11339689; doi:10.3389/fpsyg.2024.1421707)
Supplement: Supplementary file 1 [file Table_1.docx]

Supplementary Material

**Supplementary Table S1.** Instructions for the task and debriefing statement

| **Instructions for training and experimental trials**  To complete the experiment you will need to find a quiet space and stable internet connection. Please maximize your browser and make sure you can complete the experiment without distractions. It will take you around 15 minutes to complete the experiment. Click the spacebar to continue.  You will now see a woman’s face five times. We will ask you to rate each face twice, on a scale from 1 to 7. First, we want you to rate the intensity of her expression: 1 being not intense at all (e.g. a neutral expression); and 7 very intense (e.g. an extremely angry looking face). Secondly, we will ask you to rate the attractiveness of each face: 1 not attractive at all, 7 very attractive. Click the spacebar to continue.  *….. Practice trials ….*  You will now start the experimental trials. Click the spacebar to continue.  *…... Experimental trials ….* |
| --- |
| **Debriefing Statement**  Thank you for taking part in the experiment. The purpose of today's research is to investigate the relationship between eye size, attractiveness and the perceived intensity of an emotion in a face. We aim to understand whether or not the eyes size has an impact on the perceived intensity of emotions. You were asked to rate the attractiveness and the intensity of the expression of several faces. The chosen emotions for the study are some of the 6 basic emotions described by Paul Ekman (anger, joy, fear and sadness). A neutral/serious expression was added to the experiment to set a baseline for attractiveness ratings. You might have noticed that the eyes size in the images varied throughout the experiment. We are testing if eye size has an impact on how emotions are perceived. Research suggests that there is a relationship between eye size and attractiveness. In this study we are investigating whether or not there is also a relationship between eye size and perceived intensity of face expression; and attractiveness and perceived intensity of face expression. |

**Supplementary Table S2.** ANOVA for the intensity measure including the interaction with gender

| Descriptives | Neutral (Ne) | | Happiness (Ha) | | Fear (Fe) | | Sadness (Sa) | | Anger (An) | |  |  |
| --- | --- | --- | --- | --- | --- | --- | --- | --- | --- | --- | --- | --- |
|  | *Mean* | *SE* | *Mean* | *SE* | *Mean* | *SE* | *Mean* | *SE* | *Mean* | *SE* |  |  |
| Female Small eye (Sm) | 2.05 | 0.16 | 4.67 | 0.16 | 5.80 | 0.11 | 4.18 | 0.16 | 4.83 | 0.12 |  |  |
| Unchanged eye (Un) | 2.03 | 0.16 | 4.77 | 0.15 | 5.74 | 0.10 | 4.16 | 0.14 | 4.79 | 0.13 |  |  |
| Large eye (La) | 2.17 | 0.16 | 4.74 | 0.17 | 5.96 | 0.11 | 4.22 | 0.14 | 5.00 | 0.14 |  |  |
| Male Small eye (Sm) | 2.27 | 0.26 | 4.75 | 0.26 | 5.70 | 0.18 | 4.18 | 0.25 | 4.82 | 0.20 |  |  |
| Unchanged eye (Un) | 2.46 | 0.26 | 4.92 | 0.24 | 5.86 | 0.16 | 4.29 | 0.22 | 4.91 | 0.21 |  |  |
| Large eye (La) | 2.89 | 0.25 | 5.15 | 0.27 | 6.11 | 0.18 | 4.67 | 0.22 | 5.07 | 0.22 |  |  |
| Multivariate tests | Emotion | | Eye | | Emotion×eye | | Emotion×gender | | Eye×gender | | Em×eye×gender | |
|  | *p* | η_p_^2^ | *p* | η_p_^2^ | *p* | η_p_^2^ | *p* | η_p_^2^ | *p* | η_p_^2^ | *p* | η_p_^2^ |
|  | **.001*** | **.775^†^** | **.001*** | **.221^†^** | .680 | .011 | .493 | .013 | **.010*** | .085 | .464 | .016 |
| Main effects for emotion | Contrasts *(p-value)* | | | | | | | | | | Multivariate test | |
| Contrast → | Ne-Ha | Ne-Fe | Ne-Sa | Ne-An | Ha-Fe | Ha-Sa | Ha-An | Fe-Sa | Fe-An | Sa-An | *p* | η_p_^2^ |
|  | **.001*** | **.001*** | **.001*** | **.001*** | **.001*** | **.001*** | .627 | **.001*** | **.001*** | **.001*** | **.001*** | **.913^†^** |
| Single effects for eye | Female *(p-value)* | | | Multivariate test | | Male *(p-value)* | | | Multivariate test | |  |  |
| Contrast → | Sm-Un | Sm-La | Un-La | *p* | η_p_^2^ | Sm-Un | Sm-La | Un-La | *p* | η_p_^2^ |  |  |
|  | .831 | .085 | **.026*** | .086 | .083 | **.026*** | **.001*** | **.001*** | **.001*** | **.246^†^** |  |  |

*Note.* SE: standard error. η_p_^2^: partial eta squared. *Bold: significant parameter. †Bold: relevant effect size (η_p_^2^ > 0.10).

**Supplementary Table S3.** ANOVA for the attractiveness measure

| **Measure: attractiveness** | Neutral (Ne) | | Happiness (Ha) | | Fear (Fe) | | Sadness (Sa) | | Anger (An) | |  |  |
| --- | --- | --- | --- | --- | --- | --- | --- | --- | --- | --- | --- | --- |
| Descriptives | *Mean* | *SE* | *Mean* | *SE* | *Mean* | *SE* | *Mean* | *SE* | *Mean* | *SE* |  |  |
| Eye Small (Sm) | 2.28 | 0.13 | 3.23 | 0.17 | 2.35 | 0.12 | 2.35 | 0.11 | 2.50 | 0.12 |  |  |
| Unchanged (Un) | 2.90 | 0.13 | 3.87 | 0.18 | 2.73 | 0.13 | 2.77 | 0.12 | 2.78 | 0.13 |  |  |
| Large (La) | 2.80 | 0.13 | 3.77 | 0.15 | 2.62 | 0.12 | 2.70 | 0.13 | 2.81 | 0.13 |  |  |
| Tests of within-subjects | Emotion | | Eye | | Emotion-by-eye | |  |  |  |  |  |  |
|  | *p* | η_p_^2^ | *p* | η_p_^2^ | *p* | η_p_^2^ |  |  |  |  |  |  |
|  | **.001*** | **.439^†^** | **.001*** | **.402^†^** | **.001*** | .063 |  |  |  |  |  |  |
| Single effects for emotion | Contrasts *(p-value)* | | | | | | | | | | Multivariate test | |
|  | Ne-Ha | Ne-Fe | Ne-Sa | Ne-An | Ha-Fe | Ha-Sa | Ha-An | Fe-Sa | Fe-An | Sa-An | *p* | η_p_^2^ |
| Into eye Small | **.001*** | .397 | .315 | **.006*** | **.001*** | **.001*** | **.001*** | .933 | **.019*** | **.010*** | **.001*** | **.508^†^** |
| Unchanged | **.001*** | .053 | .105 | .108 | **.001*** | **.001*** | **.001*** | .497 | .470 | .896 | **.001*** | **.568^†^** |
| Large | **.001*** | **.042*** | .276 | .876 | **.001*** | **.001*** | **.001*** | .308 | **.004*** | .089 | **.001*** | **.636^†^** |
| Single effects for eye | Contrasts *(p-value)* | | | Multivariate test | |  |  |  |  |  |  |  |
|  | Sm-Un | Sm-La | Un-La | *p* | η_p_^2^ |  |  |  |  |  |  |  |
| Into emotion Neutral | **.001*** | **.001*** | .164 | **.001*** | **.448^†^** |  |  |  |  |  |  |  |
| Happiness | **.001*** | **.001*** | .202 | **.001*** | **.481^†^** |  |  |  |  |  |  |  |
| Fear | **.001*** | **.002*** | .097 | **.001*** | **.246^†^** |  |  |  |  |  |  |  |
| Sadness | **.001*** | **.001*** | .261 | **.001*** | **.343^†^** |  |  |  |  |  |  |  |
| Anger | **.001*** | **.001*** | .626 | **.001*** | **.347^†^** |  |  |  |  |  |  |  |

*Note.* SE: standard error. η_p_^2^: partial eta squared. *Bold: significant parameter. †Bold: relevant effect size (η_p_^2^ > 0.10).

## Supplementary Figures

**Supplementary Figure 1.** The classification into the groups of age (young and middle) was based on the median (percentile 50) in the study. Young age: 18 to 25 years. Middle age: 26 to 35 years.
